# GGA and GGA + U Study of ThMn_2_Si_2_ and ThMn_2_Ge_2_ Compounds in a Body-Centered Tetragonal Ferromagnetic Phase

**DOI:** 10.3390/molecules27207070

**Published:** 2022-10-20

**Authors:** Abdul Ahad Khan, Zeshan Zada, Ali H. Reshak, Muhammad Ishaq, Sabeen Zada, Muhammad Saqib, Muhammad Ismail, Muhammad Fazal-ur-Rehman, Ghulam Murtaza, Shafqat Zada, Muhammad M. Ramli

**Affiliations:** 1 Department of Physics, University of Peshawar, Peshawar 25120, Pakistan; 2 Beijing National Laboratory for Condensed Matter Physics, Institute of Physics, Chinese Academy of Sciences, Beijing 100190, China; 3 Physics Department, College of Science, University of Basrah, Basrah 61004, Iraq; 4 Center of Excellence Geopolymer and Green Technology (CEGeoGTech), University Malaysia Perlis, Kangar 01007, Perlis, Malaysia; 5 Department of Instrumentation and Control Engineering, Faculty of Mechanical Engineering, CTU in Prague, Technicka 4, 616607 Prague, Czech Republic; 6 Electrical Engineering Department, COMSATS University Islamabad, Islamabad 45550, Pakistan; 7 Department of Chemistry, Women University Swabi, Swabi 23430, Pakistan; 8 Department of Electrical and Computer Engineering, Abbottabad Campus, COMSATS University Islamabad, Abbottabad 22060, Pakistan; 9 Department of Chemistry, Lahore Garrison University, Lahore 54000, Pakistan; 10 Materials Modeling Lab, Department of Physics, Islamia College University, Peshawar 25120, Pakistan; 11 Department of BioChemistry, Quaid azam University, Islamabad 45320, Pakistan

**Keywords:** magnetic properties, ThMn_2_Si_2_, ThMn_2_Ge_2_, ferromagnetic phase

## Abstract

Our study used the full-potential linearized augmented plane waves (FP-LAPW) method to conduct a first-principles evaluation of the structural, electronic, and magnetic properties of ThMn_2_X_2_ (X = Si and Ge) compounds. To establish theoretical dependability with the currently available experimental results, computations for the structural findings of ternary intermetallic thorium (Th)-based compounds were achieved using the generalized gradient approximation in the scheme of Perdew–Burke–Ernzerhof (PBE–GGA) potential, while the generalized gradient approximation plus the Hubbard U (GGA + U) approach was employed to improve the electrical and magnetic properties. In contrast with both the paramagnetic (PM) and antiferromagnetic (AFM) phases, the ThMn_2_X_2_ compounds were optimized in a stable ferromagnetic (FM) phase, which was more suited for studying and analyzing magnetic properties. The electronic band structures (BS) and the density of state (DOS) were computed using the two PBE–GGA and GGA + U approximations. The thorium (Th)-based ThMn_2_X_2_ compound has full metallic character, due to the crossing and overlapping of bands across the Fermi level of energy, as well as the absence of a gap through both spin (up and down) channels. There was a significant hybridization between (Mn-*d* and (X = Si and Ge)-*p* states of conduction band with Th-*f* states in the valence band. The total magnetic moment of ThMn_2_Si_2_ in the ferromagnetic phase was 7.94534 μB, while for ThMn_2_Ge_2_ it was 8.73824 μB with a major contribution from the Mn atom. In addition, the ThMn_2_Ge_2_ compound’s total magnetic moment confirmed that it exhibits higher ferromagnetism than does the ThMn_2_Si_2_ compound.

## 1. Introduction

Interest in the search for intermetallic phase materials has been rising over the last few decades because of their mysterious features and the diverse range of sensible and modern applications, such as the computer readings by shape memory alloys (SMAs) in the jewelry and dentistry industries [1,2], as well as the rapid utilization of intermetallic materials for polishing various attractive and intriguing giant magneto resistive (GMR) and colossal magneto resistive (CMR) materials. Magnetoresistance inside metallic thin films varies and regulates resistivity, due to the presence of an external magnetic field to progress the technology [3]. To date, the achieved applications of GMR materials have been uses as spin valves, as spin filters collectively with magnetic field dependent sensors [4,5] (in which the most significant and critical part is the GMR material that is further used to deal with read heads in modern hard disk drives), in several biosensors, and in advanced micro-electro mechanical (MEMS) systems. These intermetallic materials have a distinct electronic structure and deserve special attention; if further investigated, they could lead to a variety of novel quantum characteristic features.

Normally, intermetallic phases with the formula AM_2_X_2_ (where A belongs to rare/alkaline earth metals), M belongs to d-block metals), and X represents Group 13–15 elements) are typically found in various structural types, such as the body-centered tetragonal of space groups ThCr_2_Si_2_ and CeAl_2_Ge_2_ (14/mmm) and CaAl_2_Si_2_ (P$\bar{3}$m1), respectively. In numerous works, the latter group, in particular, was utilized to investigate various physical aspects of intermetallic ternary compounds [6,7,8,9,10].

The discovery of several isostructural thorium-based ThM_2_X_2_, (M = Cr, Mn, Fe, Co, Ni, and Cu; X = Si or Ge) ternary intermetallic silicide and germanide compounds that crystallize in the tetragonal CeAl_2_Ge_2_-type structure at temperatures ranging from 100 K to 570 K by means of the Faraday method [11,12] was reported early in 1971, wherein all three components can be replaced while maintaining the same structure. Furthermore, all of these compounds have ordered BaA1_4_-type crystal structures. In contrast to the pure elements, the transition metal atoms in the crystal structure are spaced much farther apart by X-Th-X sandwiches, implying that metamagnetic activity is expected [13]. Depending on the atomic number of the attached transition metal atoms, the thorium containing silicide and germanide compounds were found to be ferromagnetic or antiferromagnetic.

Omejec and Ban [13] were the first to study the magnetic properties of these compounds experimentally. They discovered a maximum of magnetic susceptibility of approximately 500 K in ThMn_2_Si_2_, but a small saturation magnetization below 400 K in ThMn_2_Ge_2_. They claimed that by comparing inverse susceptibility curves of ThM_2_X_2_ compounds with Curie–Weiss law curves for magnetic susceptibility measurement, they were able to determine “a complete magnetic parameter, numeric values for Neel and Curie temperatures, along with the effective magnetization per atom of transition metal”. They further noted the ferromagnetic ordering at positive Curie temperatures in the thorium compound ThMn_2_X_2_ [13]. Because thorium (Th) has no magnetic dipole moment, the ordering is caused by 3d-transition elements.

Magnetic studies on Mn-containing compounds were carried out to see how changing the Mn–Mn distance affects magnetic ordering, Curie temperature, and anisotropy [11,12]. The first-principles approach and XPS studies was used to examine the magnetic structures and magnetic phase transitions in intermetallic-layered RMn_2_Si_2_ compounds [14,15]. In accordance with experimental results, it was discovered that the distances between the Mn ions in the ab plane, as opposed to the interplane distances, influence the type of ordering of Mn magnetic moments along the c-axis [14].

Over a wide temperature range (4.2 K to 1200 K)., the magnetic properties of RMn_2_Ge_2_ and RMn_2_Si_2_ compounds were examined. The Curie temperatures were found to be above 300 K when rare earth (R) was replaced with La, Ce, Pr, and Nd, and the easiest direction of magnetization was discovered to be along the c-axis [16]. When R was replaced with a heavy rare earth, an antiferromagnetic coupling between the R and manganese moments was observed at about 4.2 K, whereas a ferromagnetic coupling was observed when R was replaced with a light rare earth. At 4.2 K, YMn_2_Si_2_, ThMn_2_Si_2_, and ThMn_2_Ge_2_ all had a small moment at less than 0.2 µB [16]. Furthermore, by using the self-consistent Korringa–Kohn–Rostoker approach, the electronic structure of the tetragonal RMn_2_Ge_2_ (R = Ca, Y, La, and Ba) antiferromagnets was reported by a few researchers. All of the reported Mn-based compounds had computed magnetic moments that were in good accord with the neutron data. All systems were metallic, according to antiferromagnetic density of states, although ΒaΜn_2_Ge_2_ was discovered to be close to the semi-metallic limit [17].

The magnetic properties of the RMn_2_Ge_2_ compounds have been studied were between the temperature range 4.2 and 500 K. Only R = La, Ce, Pr, and Nd are ferromagnetic, with Curie temperatures of 306, 316, 334, and 334 K, respectively [18]. Until very low temperatures are reached, the heavier rare-earth compounds do not arrange ferromagnetically. In both magnetization and resistivity-vs-temperature measurements, GdMn_2_Ge_2_ exhibits an abrupt magnetic transition at 97 K. It is hypothesized that the Mn moments in this compound order antiferromagnetically among themselves above 97 K, whereas the Gd moments are disordered. Mn moments pair ferromagnetically with each other below 97 K, but they oppose the ordered Gd sublattice [18]. An antiferromagnetic interaction between Mn and rare-earth spins can explain the magnetic moment reported at 4.2 K for all of the investigated compounds. McCall et al. [19] studied the RC0_2_Ge_2_ compounds for the first time and determined that cobalt has no moment in these Ge rare-earth-based compounds. It is important to select a transition metal with a less fully filled d band in order for M (transition metal) atoms to have a moment. According to Gyorgy et al. [20], the Sm-based SmMn_2_Ge_2_ compound is ferromagnetic below a Curie temperature of T c = 350 K. Further cooling causes an antiferromagnetic phase, T = 150 K, to form, which is almost stable until T = 100 K is reached, when it transitions to a ferromagnetic phase.

In line with the preceding discussion, several researchers have tried to physically explore the physical structure and magnetic properties of ThMn_2_X_2_ (X = Si, Ge) compounds to some extent, although theoretical evidence is still lacking. Because of their attracting physical properties, some research has already been done on members of the same class of materials [21,22,23,24,25,26]. The current study was prompted in part by a knowledge gap in the fundamental structural, electronic, and magnetic properties of ternary intermetallic ThMn_2_Si_2_ and ThMn_2_Ge_2_ compounds, which were not well known or improved, in comparison with their counterparts.

## 2. Computational Details

Our investigations for ThMn_2_X_2_ (X = Si and Ge) compounds were performed using the full-potential linearized augmented plane wave (FPLAPW) method and the Wien2 k algorithm [27]. Muffin-tin sphere radii (RMT) were chosen, so that the atoms did not overlap at values of 2.5 atom units (a.u.) for thorium (Th), 2.41 and 2.08 a.u. for manganese (Mn), and 1.78 and 1.98 a.u. for silicon (Si) and germanium (Ge), respectively. The Muffin-tin minimum radius (RMT) and the greatest possible reciprocal lattice constant, Kmax, were utilized to determine the cut-off parameter for the basis set’s convergence. RMT*Kmax = 7 illustrates the separation among various valence and core states. This tactic relies on the partitioning of the entire crystal into the non-overlapping muffin-tin (Mu-T) spheres that are parted from one another by an interstitial zone. This is where spherical harmonic functions within MT spheres are supported by a simple basic set function that is chosen and extended, and basic plane waves are also used in the interstitial area. The biggest vector (Gmax) in the charge density’s Fourier expansion has a magnitude of 12 atom units (a.u.)^−1^ [28,29,30,31]. We employed energy convergence with the value 0.0001 Ry and charge convergence with the value 0.001 e for the self-consistency cycle. We did not factor in the impact of spin orbit coupling in our estimates. The tetrahedron approach [32], with special 195 k-points (together with 10 × 10 × 10 k-points in the complete BZ) in the irreducible wedge, was taken into consideration to determine the charge density in every self-consistency step for the integration of the Brillouin zone (BZ). Furthermore, with both the exchange and the correlation (GGA + U/GGA–PBE/LDA) potentials [33,34,35,36] that are implemented in Wien-2 k code, the structural characteristics of the compound ThMn_2_X_2_ (X = Si and Ge) were determined. According to previously published research [37], the value employed in this study for both compounds is U = 6 eV, which is a fairly excellent approximation for handling *d*-states of Mn atoms. GGA–PBE and GGA + U potentials were used to find the electronic and magnetic characteristics of ThMn_2_X_2_ (X = Si and Ge) compounds.

## 3. Results and Discussion

### 3.1. Structural Properties

**Figure 1:**
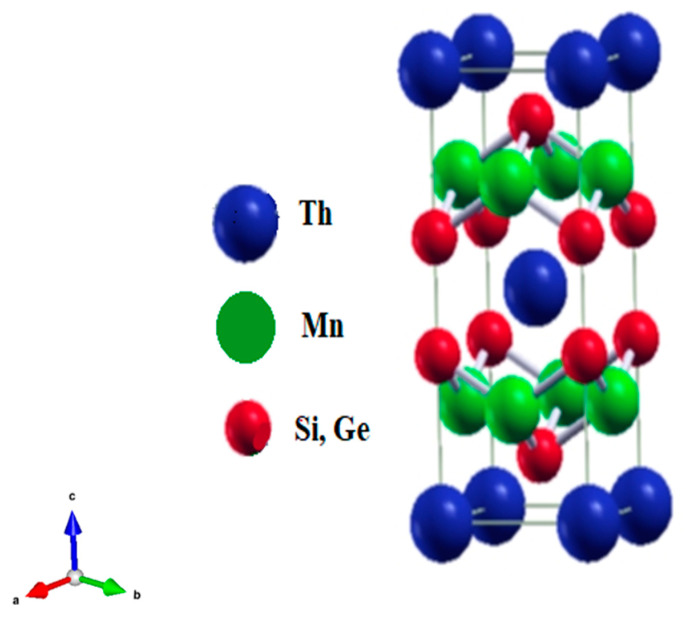
Relaxed crystal structure of ThMn_2_Si_2_ compound.

The thorium (Th)-based isostructural ternary intermetallic silicide and germanide systems, along with general formula ThMn_2_X_2_ (X = Si and Ge), has crystallized in the body-centered tetragonal lattice structure with the space group (14/mmm #139). The site positions for thorium (Th) atoms are the Wyckoff position 2(a) [38]; for manganese (Mn) atom, it is 4(d); for (X = Si and Ge) silicon and germanium atoms, it is 4(e). As a result, atoms of the same element are stacked in the sequence Th-X-Mn-X-Th on alternating layers that are stacked along the c-axis, as shown in Figure 1.

The structural morphology of the compounds is determined by computing both the lattice atomic positions and cell dimensions. The ferromagnetic phase is the most suited and stable phase for ThMn_2_X_2_ (X = Si, Ge), based on optimization. In addition, available experimental results, up to our literature review, concluded that the most appropriate phase for these materials is the ferromagnetic phase [13,16].

**Figure 2:**
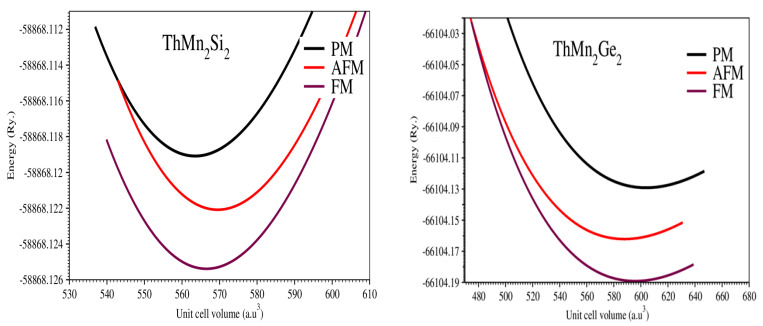
Optimized plots of ThMn_2_Si_2_ and ThMn_2_Ge_2_ compounds.

We estimated the total energy for dissimilar volumes in the vicinity of the calculated experimental volume to optimize the unit cell and to obtain the ground state energy. To derive the compound’s ground state parameters, we reported its total energy and plotted it versus volume, fitting it to the known empirical Murnaghan’s equation of state (EOS) [39]. Figure 2 depicts the computed total energy of the state as a function of volume in the paramagnetic (PM), ferromagnetic (FM), and antiferromagnetic (AFM) phases for compounds ThMn_2_Si_2_ and ThMn_2_Ge_2_.

Only PM phase energy is provided for both of these reported compounds; the remaining parameters were calculated by PBE–GGA potentials and derived in the FM phase, as in a prior study [31]. Furthermore, in the FM phase, both compounds were stabilized. Table 1 shows the computed values of the lattice parameters, the ground state energy, the unit cell volume, the bulk modulus, and the derivative of the bulk modulus for both compounds, based on structural optimization.

When the Si anion was replaced by Ge, the lattice constants (ɑ and c(Å)) and unit cell volume ($V_{0}$) increased as the PM and FM phases were computed. This improvement was due to an increase in the atomic sizes of the anions as they move from the Si to the Ge element. Finally, the B(GPa), which describes the natural compressibility of materials, showed a definite declining trend in the computed values from Si to Ge, making the ThMn_2_Ge_2_ compound more squeezable or compressible than the ThMn_2_Si_2_ compound.

### 3.2. Electronic Properties

#### Band Structures and Density of States

**Figure 3:**
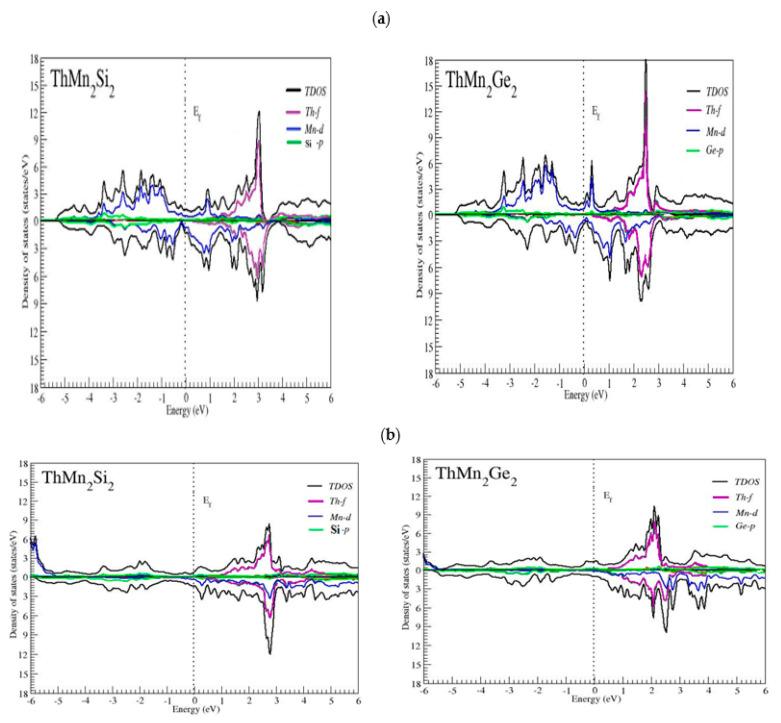
(**a**) Plotted DOS of ThMn_2_Si_2_ and ThMn_2_Ge_2_ compounds in body-centered tetragonal ferromagnetic phase, using GGA in both orientations; (**b**) plotted DOS of ThMn_2_Si_2_ and ThMn_2_Ge_2_ compounds in body-centered tetragonal ferromagnetic phase, using GGA + U in both orientations.

Electronic band structure and density of state are important factors in determining crystal structure [40,41,42,43]. The evaluations of the total density of states (TDOS) and partial density of states (PDOS) are particularly important for a full understanding of a compound’s bonding character. In the energy range between −6 eV and 6 eV, we estimated the TDOS and the PDOS using two distinct (exchange and correlation) known approximations, PBE–GGA and GGA + U. For spins both up and down the channels, Figure 3a,b depicts the DOS plots of the thorium (Th)-based ThMn_2_Si_2_ and ThMn_2_Ge_2_ compounds.

Both compounds have a full metallic character, as the total and partial (*p*, *d*, *f*) states appear to cross the Fermi energy level in both spin states. As a result, we concluded, as shown in Figure 3a,b, that the contribution of orbitals in both compounds is relatively similar. Furthermore, it was obvious from the TDOS that the active progress at (EF) was primarily because of the Mn-*d* state. Moreover, in the region of valence band, there was a substantial hybridization between the Mn-*d* and X = Si and Ge-*p* states, with the Th-*f* state in the region of the conduction band. These states bore complete responsibility for the materials with metallic characteristics.

The Mn-*d* state’s primary appearance and contribution were placed at the Fermi level, as well as the leading spread ranging from high to low energy with values and approximations (−0.2 to −4 eV through (GGA–PBE) and −4.9 to −6 eV through (GGA + U)) for ThMn_2_Si_2_ while (0 to −3.4 eV through (GGA–PBE) and −5 to −6 eV through (GGA + U)) for ThMn_2_Ge_2_ in the region of valence band is clearly observed. This change toward a lower energy range of *d*-state for ThMn_2_X_2_ was due to the application of (GGA + U), which handled the Mn atom-localized *d*-shell electrons. Furthermore, the elimination of the Mn-*d* state from both channels at lower energies when employing (GGA–PBE) and the shift towards higher energies of the identical Mn-*d* state by down spin when using (GGA + U) are visible in the DOS plots of Figure 3 a,b. In addition, the Th-*d* state with a straight line emerges throughout the full energy range of the valence band area, with a minor contribution from the (X = Si and Ge)-*p* state in the region of the valence band and over the whole energy range in ThMn_2_X_2_, through both approximations. The value employed in this study for both compounds was U = 6 eV, which is a fair estimate for dealing with Mn atom *d*-states, as stated in a previous study [37].

Furthermore, in the conduction band region across both spin states, the Th-*f* state showed pointedly toward with values that were higher in the energy range: 0.8 eV to 3.4 eV through both potentials for ThMn_2_Si_2_ and 0.6 eV to 3 eV (GGA–PBE) and 0.4 eV to 2.8 eV (GGA + U) for the ThMn_2_Ge_2_ compound. Furthermore, the Mn-*d* state placed at the Fermi level made a minor contribution in the different spin states of ThMn_2_X_2_ toward lower energy by GGA–PBE and toward higher energy by GGA + U approximation. To conclude the extremely symmetrical or balanced appearance of ThMn_2_X_2_, various states’ spectra (Th-*f*, Mn-*d* and X-*p*) through both spin states resided in bands of both the valence and the conduction region, indicating that Stoner’s argument was frequently fulfilled [44,45].

**Figure 4:**
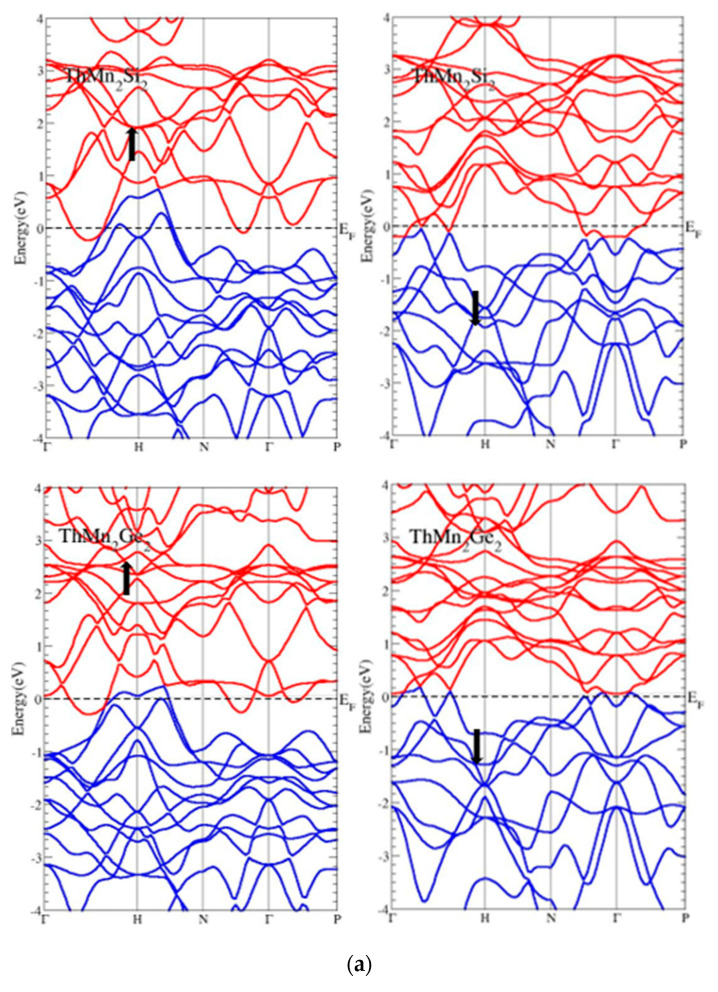

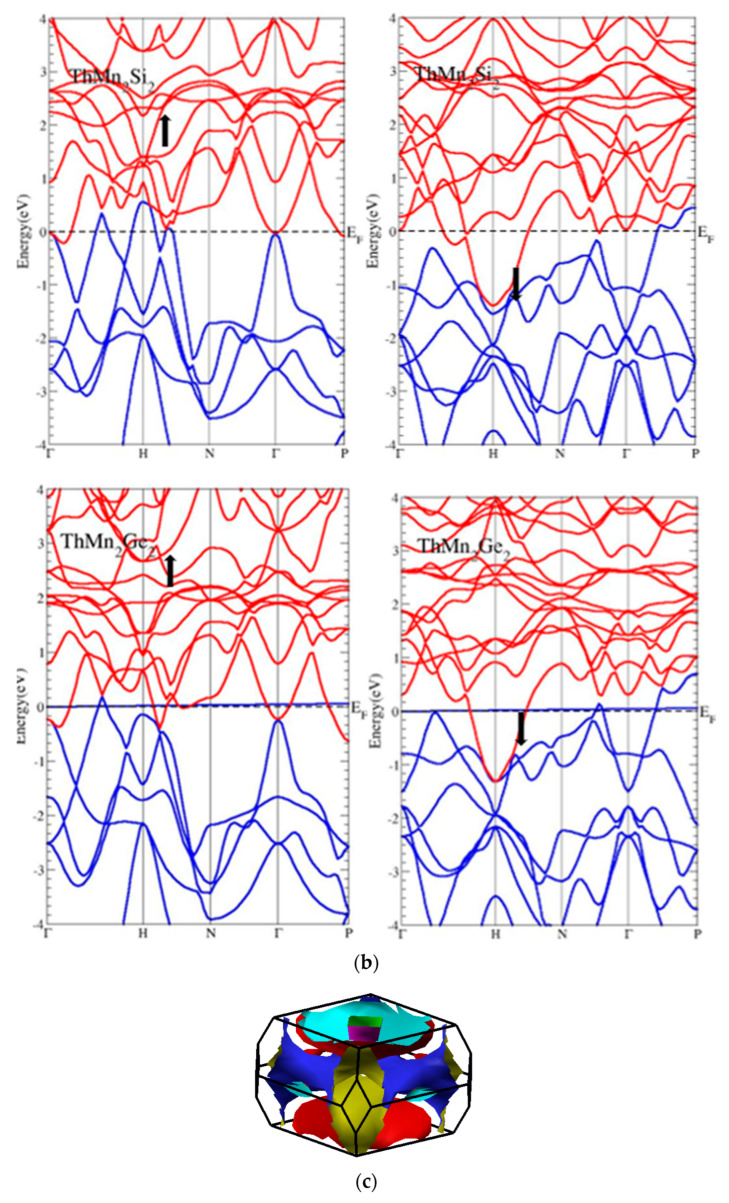
(**a**) Band structures of the ThMn_2_Si_2_ and ThMn_2_Ge_2_ compounds in body-centered tetragonal ferromagnetic phase while using GGA–PBE in both spin states; (**b**) band structures of the ThMn_2_Si_2_ and ThMn_2_Ge_2_ compounds in body-centered tetragonal ferromagnetic phase while using GGA + U in both spin states; (**c**) Fermi surface sheet of ThMn_2_Ge_2_ as a prototype of ThMn_2_X_2_, in the body-centered tetragonal Brillouin zone boundaries. The character of Fermi surfaces are primarily constituted by the Th *f*, Mn *d*, and X *p* states.

To further understand the electronic characteristics of ThMn_2_X_2_ (X = Si, Ge), the electronic band structure is displayed in Figure 4a,b for both spin states, using two distinct approximations, PBE–GGA and GGA + U. The Fermi level of energy was set to origin as a reference point in these plotted figures, and the analyzed band structures in the Brillouin zone (BZ) are displayed in Figure 4b, while the calculated Fermi surface of ThMn_2_Ge_2_ as a prototype (shown in Figure 4c) along the Γ, H, N, and (Γ, P) high symmetry directions are also shown. Furthermore, the minima of CB were more dominant because of Mn-*d* and Th-*f*, while the VB maxima were mostly due to *p* characters from the Si/Ge in both spin channels. However, because more continuing *d* orbitals arise in compounds various channels, the overlap between the *p* (Si and Ge) and *f* (Th) with *d* (Mn) orbitals twists to the stronger side. As a result, the overlaps between these bands in the compounds under study were sufficient for metallicity.

It is easy to notice the valence band maximum’s considerable dispersion at the high-symmetry (H) point. By utilizing PBE–GGA and GGA + U approximations with small adjustments in the features, the bands with dominant shoulders appeared on both sides while crossing the Fermi energy level and remained in the region of the conduction band, especially in the up-spin states of ThMn_2_X_2_. This was similar to the disappearance of the same single band at the H point in the spin-down channel of the ThMn_2_Si_2_ compound and the minor appearance of a single narrow band dispersion among the high symmetry (Γ and H, Γ and N, Γ and P) basal points in the compound ThMn_2_Ge_2_ spin-down state. In addition, the conduction band minimum for the ThMn_2_X_2_ compounds exhibited the same pattern of dispersion of a few bands between the high symmetry points (Γ and H, Γ and N, Γ and P) from both spin states, indicating that the conduction bands held additional electrons and were fully filled, as well as meeting and overlapping the mechanisms with the available valence bands by both (PBE–GGA and GGA + U) approximations.

The bands clearly split when the GGA + U approximation was computed; however, both thorium compounds continued to show their metallic nature. The CB traveled up and the VB traveled down at high symmetry (Γ point) by bounding *d*-states and computing the approximation GGA + U. Furthermore, the calculation using the GGA + U resulted in peculiar behavior, such as the absence of gaps in the ThMn_2_X_2_ compounds where the valence and conduction bands met at both high Γ symmetry points in up-spin channels while shifting the same meeting bands back to their original locations in spin-down channels.

In addition, there was no energy (absent) gap at the Femi level of energy, expressing the pure metallic nature, although the spin-up channel states were significantly closer to one another than the spin-down channels that were nearby the Fermi level. Even the closer states allowed for simple electron mobility, which over time affected the materials’ conductivity. Moreover, the compounds adopted the tetragonal ThCr_2_Si_2_-type structure with three-dimensional electron and hole-like multiband Fermi surfaces (FSs), as appeared in the majority of the same types [46,47] and other types [41,48,49] of compounds that were related to the phonon-mediated types that were currently under electron-phonon mediated superconductivity, according to a few other research studies that calculated Fermi surfaces (FSs). Due to the crossing and overlapping of bands across the Fermi level of energy, as well as the absence of a gap in both spin states when utilizing two separate (PBE–GGA and GGA + U) approximations, the thorium compound ThMn_2_X_2_ band structures generally described the full metallic nature.

### 3.3. Magnetic Properties

The spin polarization calculations using the PBE–GGA and GGA + U potentials were computed to explore the magnetic characteristics of the ThMn_2_Si_2_ and ThMn_2_Ge_2_ compounds. Table 2 summarizes the overall **m^c^** magnetic moment (MM) of each cell, interstitial and local (Th, Mn, Si, and Ge) individual (MM) magnetic moments of the materials under study. According to the noted data, the active and most contributing atom in the total cell magnetic moment (MM) of ThMn_2_X_2_ (X = Si and Ge) is the Mn, contributing high values of a parallel ferromagnetic nature, in contrast to other atoms such as those at the interstitial and individual Th, Si and Ge atoms that were participating with negative anti-parallel values by both PBE–GGA and GGA + U approximations.

The fundamental source of magnetization in these thorium (Th) ternary intermetallic materials predominantly originates from unoccupied or empty Th-*f* and Mn-*d* orbitals. However, except for the leading manganese site, the remaining sites had a tendency to lower the ferromagnetic nature of the materials under study. These thorium ternary intermetallic compounds’ fundamental source of magnetism, in particular, derived from unoccupied or empty Th-*f* and Mn-*d* orbitals. As a result, with the exception of the dominating manganese site, the remaining spots tended to reduce the overall ferromagnetic character of the aforementioned compounds.

The stable or steady magnetic ground state, as shown in optimization plots in Figure 2, was ferromagnetic, as previously reported in experimental works [13,16]. This is supported by the non-negative or positive values of the net magnetic moments (MM) of the ThMn_2_Si_2_ and ThMn_2_Ge_2_ compounds by both the PBE–GGA and GGA + U approximations. Due to the compounds’ ferromagnetic nature, both in this study and in earlier research [13,16,21,22,28,30,31], the parallel magnetic moment was the primary focus of our work. Furthermore, the calculation due to the GGA + U approximation provided a value with a higher scale or magnitude, compared with other currently known potentials, as it frequently dealt with d-shell-localized electrons of the Mn atom and raised its MM to some level in comparison with the PBE–GGA approximation. In addition, the calculation due to the GGA + U yielded a value with a larger magnitude, compared with other approximations that were previously identified, because it primarily dealt with *d*-shell-localized electrons of the Mn atom and raised its MM to a greater extent than the PBE–GGA.

In addition, the computed values of ThMn_2_X_2_ (MM) by both approximations, precisely at interstitial and individual Th, Si, and Ge atoms, opposed the net magnetic moment, whereas Mn supported it due to the dominant appearance of the Mn-*d* state over the entire range of energy in both spin states, as previously mentioned, whereas investigating by both approximations, as shown in the DOS plots Figure 3a,b, supported this claim. The antiferromagnetic (AFM) interaction between the electrons in the valence band (VB) was seen by the opposing sign appearing in the magnetic moments of the Inst, Th, Si/Ge, total cell, and Mn element. Due to Mn being oriented in the direction of the compounds’ net magnetic moment, ThMn_2_Ge_2_ exhibited higher ferromagnetism. As seen in Table 2, the value of M^Mn^ changed from 2.17623 to 4.42327 when the d states of the Mn atom were bound through the GGA + U potential. Finally, the total magnetic moments aligned in parallel by both the PBE–GGA and GGA + U potentials, indicating that the thorium (Th)-based ThMn_2_Ge_2_ metallic compound exhibited stronger FM behavior than the ThMn_2_Si_2_ compound, due to a difference of electro negativity between available Ge (2.01) and Si (1.90) atoms in a prior study [23,24,25]. As a result, the greater the electronegativity difference, the greater the magnetic moment demonstrated by the ThMn_2_Ge_2_, confirming stronger ferromagnetism.

## 4. Conclusions

We used the full-potential linearized augmented plane wave (FPLAPW) method through DFT within the generalized gradient approximation (GGA), as applied in the Wien2 k code, to investigate the various physical properties of tetragonal structure thorium (Th)-based ThMn_2_X_2_ (X = Si and Ge) compounds. When the silicon (Si) anion was replaced by germanium (Ge), the lattice constants (ɑ and c(Å)) and unit cell volume ($V_{0}$) increased due to the increase in the atomic sizes of the anions as the PM and FM phases were computed. Furthermore, the B(GPa), which describes the natural compressibility of materials, showed a definite declining trend in computed values from Si to Ge, making the ThMn_2_Ge_2_ compound more squeezable or compressible than ThMn_2_Si_2_. While we employed the GGA + U approach to enhance the electrical and magnetic properties, the computations for the structural findings of ternary thorium intermetallic were produced using the PBE–GGA potential to establish theoretical reliability with the currently existing experimental works. The ThMn_2_X_2_ were optimized in a ferromagnetic stable phase, which was better suited than paramagnetic and antiferromagnetic phases for studying a compound’s magnetic properties. The plotted crossing and overlapping electronic band structures (BS) (with absent gap) and the density of state (DOS) plots by both spin states indicated that the ThMn_2_X_2_ compound had full metallic character, due to the presence of significant hybridization between (Mn-d and (X = Si and Ge)-*p* states with Th-*f* states. The total magnetic moment of ThMn_2_Si_2_ in the ferromagnetic phase was 7.94534 μB, while the total magnetic moment for ThMn_2_Ge_2_ was 8.73824 μB (exhibiting higher ferromagnetism), with a major contribution from the Mn atom via the GGA + U approach. Additionally, the achieved ferromagnetism in the examined compounds was revealed by the acquired total magnetic moments (MM), which were aligned in parallel to the overall ferromagnetic direction.

## Figures and Tables

**Table 1:** Noted unit cell parameters of ThMn_2_Si_2_ and ThMn_2_Ge_2_ compounds, together with available experimental studies.

Compounds	Lattice Constant (A)^0^		V_0_ (a.u)^3^	B (GPa)	Bp	E_0_ (Ry)		
	a	c				FM	PM	AFM
**ThMn_2_Si_2_** Exp ^b^	4.015 4.019 ^b^	10.480 10.483 ^b^	563.5188	152.9576	5.0	−58,868.125	−58,868.119	−58,868.122
**ThMn_2_Ge_2_** Exp^a,b,c^	4.088 4.084 ^a^, 4.090 ^b^, 4.690 ^c^	10.904 10.930 ^a^, 10.907 ^b^, 10.907 ^c^	595.9810	118.4229	5.44	−66,104.19	−66,104.13	−66,104.16

^a^ Ref. [11], ^b^ Ref. [16], ^c^ Ref. [18].

**Table 2:** Magnetic moments of the interstitial region (m^inte^), single atoms (**M^Th/Mn/Si/Ge^**), and overall cell for the ThMn_2_Si_2_ and ThMn_2_Ge_2_ compounds computing both schemes (Bohr magnetrons μ_B_).

Compounds	m^inte^	M^Th^	M^Mn^	M^Si/Ge^	m^cell^	Δ = m^cell^ − M^Mn^
ThMn_2_Si_2_(PBE-GGA)	−0.04156	−0.00300	1.90730	−0.04194	3.68615	1.77885
GGA + U	−0.15905	−0.08126	4.18022	−0.08740	7.94534	3.76512
Exp.	-----------	-----------	2.98 ^a^	-----------	-----------	-----------
Other calc.	-----------	-----------	-----------	-----------	-----------	-----------
ThMn_2_Ge_2_(PBE-GGA)	−0.17642	−0.05845	2.17623	−0.03884	4.03992	1.86369
GGA + U	0.01043	−0.02784	4.42327	−0.04544	8.73824	4.31497
Exp.	-----------	-----------	1.57 ^a^, 0.10 ^b^	-----------	-----------	-----------
Other calc.	-----------	-----------	-----------	-----------	-----------	-----------

^a^ Ref. [13], ^b^ Ref. [16].

## Data Availability

The data presented in this study are available on request from the corresponding author.

## Sample Availability

Samples of the compounds are not available from the authors.
